# Quantitative CT features for COPD classification and GOLD spirometric severity assessment: a single-center model-development study

**DOI:** 10.3389/fmed.2026.1900490

**Published:** 2026-10-01

**Authors:** Kaifeng Du, Jiameng Gao, Ji Li, Xiaojing Liu, Fang Wang, Yuhao Qian, Xiwen Gao, Zhihong Chen

**Affiliations:** 1 Department of Respiratory and Critical Care Medicine, Fudan University Minhang Hospital, Shanghai, China; 2 Department of Respiratory and Critical Care Medicine, Zhongshan Hospital, Fudan University, Shanghai, China; 3 Department of Respiratory and Critical Care Medicine, Rushan People's Hospital, Rushan, China; 4 Department of Respiratory and Critical Care Medicine, The Affiliated Hospital of Qingdao University, Qingdao, China; 5 Department of Pulmonary and Critical Care Medicine, Institute of Respiratory Diseases, Shanghai Jiaotong University School of Medicine Xinhua Hospital, Shanghai, China

**Keywords:** bronchial morphology, chronic obstructive pulmonary disease (COPD), model development, quantitative CT, visible-airway dimensions

## Abstract

**Background:**

Chronic obstructive pulmonary disease (COPD) is diagnosed primarily by post-bronchodilator spirometry, but quantitative CT can characterize emphysema and visible-airway structural abnormalities that are not fully captured by airflow measurements alone. Because CT-derived emphysema and airway features have been extensively studied, this single-center study aimed to develop and describe quantitative CT-based classification models and to explore bronchial-tree morphology as a hypothesis-generating imaging phenotype, rather than to establish a clinically validated diagnostic replacement for pulmonary function testing.

**Methods:**

We enrolled 229 participants (138 with COPD and 91 control participants) at Fudan University Zhongshan Hospital. HRCT was used to quantify visible-airway dimensions at a prespecified eighth-generation airway, emphysema extent quantified as low attenuation area percentage (LAA%), and bronchial-tree morphology. Clinical variables including smoking history, modified Medical Research Council (mMRC) dyspnea score, and pulmonary function tests were recorded. Multivariable logistic regression was used to develop single-center models for COPD classification and for classification of severe airflow obstruction (FEV1%pred < 50%, corresponding to GOLD spirometric grades 3–4). Model performance is reported as apparent performance because internal and external validation were not performed.

**Results:**

Compared with control participants, COPD participants had smaller airway lumen area, higher wall-to-diameter ratio (T/D), higher wall area percentage (WA%), and higher LAA%. Mean wall thickness did not differ significantly, indicating that the group difference mainly reflected lumen narrowing rather than wall thickening. Emphysema extent was associated with lower FEV1/FVC and FEV1%pred. Sparse-type bronchial morphology was associated with COPD in multivariable analysis (OR 28.6, 95% CI 6.7–149.8), but the wide confidence interval and the need for reproducible operational validation require cautious interpretation. The combined clinical-CT development models achieved apparent AUCs of 0.906 for COPD classification and 0.859 for classification of severe airflow obstruction.

**Conclusion:**

Quantitative HRCT emphysema and visible-airway measurements were associated with COPD status and spirometric airflow obstruction. The models should be regarded as single-center development models, and the bronchial-tree morphology finding should be interpreted as hypothesis-generating until reproducibility, calibration, incremental value, and external validation are demonstrated.

## Introduction

Chronic obstructive pulmonary disease (COPD) is a common chronic airway disease characterized by high prevalence, treatment difficulty, and frequent exacerbations, posing a significant public health challenge globally (1). In routine clinical practice, COPD diagnosis is primarily based on post-bronchodilator spirometry, particularly an FEV1/FVC ratio below 0.70 according to GOLD criteria (1). However, spirometry mainly reflects global airflow limitation and may not fully capture structural heterogeneity such as emphysema distribution, visible-airway remodeling, or regional parenchymal destruction (2, 3). Quantitative CT can therefore provide complementary structural information, although it should not be considered a replacement for pulmonary function testing.

Quantitative CT measures of emphysema burden, air trapping, and airway dimensions have been widely investigated and have been associated with COPD presence, lung function, exacerbation risk, and clinical outcomes. Recent imaging-cluster and longitudinal quantitative-imaging studies have further placed CT-derived features within the broader context of COPD phenotyping and disease progression (4, 5). These studies indicate that CT-based assessment may help characterize COPD heterogeneity, but they also suggest that conventional emphysema and airway measurements are established imaging features rather than entirely novel biomarkers. In this context, further work is needed to determine whether additional morphological features, such as bronchial-tree configuration, may provide complementary information beyond conventional quantitative CT parameters. Additional COPDGene and PRM studies further support the relationship between CT emphysema/airway phenotypes, exacerbation risk, and disease progression (6–9).

In this single-center model-development study, we analyzed clinical characteristics, CT-derived visible-airway dimensions, emphysema parameters, and bronchial-tree morphology in 229 participants. The objectives were to describe CT features associated with spirometry-defined COPD, develop exploratory classification models for COPD and severe airflow obstruction, and evaluate compact/sparse bronchial-tree morphology as a hypothesis-generating imaging feature. Because the models were developed in a single-center cohort and require further validation, they are interpreted as exploratory development models rather than clinically validated diagnostic tools. Prior total-airway-count studies also suggest that reduced visible airway branch counts are associated with emphysema, clinical severity, and COPD progression, supporting the rationale for evaluating bronchial-tree morphology (10, 11).

## Materials and methods

### Study population

This single-center retrospective model-development study included 229 participants from Zhongshan Hospital, Fudan University, including 138 patients with COPD recruited from the outpatient clinic and 91 control participants recruited from the health examination center between January 2022 and December 2024. The initial clinical dataset included 400 participants; 171 were excluded because of incomplete clinical, pulmonary function, or imaging data, leaving 229 participants for the final analysis. COPD was defined according to the GOLD 2022 spirometric criteria, using a post-bronchodilator FEV1/FVC ratio <0.70. Control participants were health examination participants from the same hospital who had no clinical diagnosis of COPD and had available clinical, pulmonary function, and HRCT data.

The inclusion criteria for the control group were as follows: (1) adult participants recruited from the health examination center of Zhongshan Hospital, Fudan University; (2) absence of a clinical diagnosis of COPD; (3) availability of pulmonary function test results and HRCT images; and (4) availability of key clinical information required for the present analysis. When pulmonary function data were available, control participants were required to have no spirometric evidence of airflow obstruction, defined as post-bronchodilator FEV1/FVC ≥0.70.

The exclusion criteria were as follows: (1) incomplete clinical information, pulmonary function data, or HRCT data; (2) HRCT images unsuitable for quantitative airway or emphysema analysis because of poor image quality, incomplete lung coverage, or segmentation failure; (3) missing data required for model development; and (4) documented major pulmonary diseases other than COPD that could substantially affect pulmonary function or CT-based airway/emphysema assessment, including asthma, bronchiectasis, interstitial lung disease, active pulmonary infection or tuberculosis, lung cancer, previous lung surgery, or other clinically significant structural lung diseases.

Clinical data included smoking history, modified Medical Research Council (mMRC) dyspnea score, pulmonary function test results, HRCT findings, and St. George's Respiratory Questionnaire (SGRQ) score. Smoking exposure and respiratory symptoms were recorded for both COPD patients and control participants when available. The different recruitment sources of COPD patients and control participants may have introduced selection and spectrum bias; therefore, model performance was interpreted as apparent discrimination within this single-center cohort rather than validated diagnostic performance in a general screening or real-world diagnostic population (12–14). The participant-flow process is shown in Supplementary Figure 1. This study was approved by the Ethics Committee of Zhongshan Hospital, Fudan University (Ethics Approval No. B2019-309R), and all participants provided written informed consent.

### HRCT acquisition and quantitative analysis

HRCT images were acquired under a standardized inspiratory protocol. Participants were scanned in the supine position with both arms raised above the head using a Siemens Somatom Definition AS 32-slice, 64-layer CT scanner. Before scanning, participants were instructed to inhale deeply and hold their breath during image acquisition. The scanning range was reconstructed with a 1-mm slice thickness to obtain HRCT images. Sagittal reconstructions were subsequently performed for further assessment. Expiratory CT scans were not included in the present quantitative analysis.

Images were reviewed on a PACS workstation. Two experienced radiologists who were blinded to clinical data used Inspace software to quantify emphysema and airway parameters. Emphysema extent was quantified as the percentage of low attenuation area (LAA%), using a prespecified threshold of < −950 HU to define low attenuation lung regions. LAA% was calculated for both lungs and, where applicable, separately for the right and left lungs.

Visible-airway dimensions were measured at the eighth-generation airway. The measured parameters included wall thickness (WT), wall thickness-to-diameter ratio (T/D), wall area (WA), lumen area (LA), wall area percentage (WA%), minimum internal diameter (Din), and minimum external diameter (Dout). Because standard CT cannot directly resolve terminal small airways, these variables were interpreted as visible-airway or indirect small-airway-related CT indices rather than direct measurements of terminal bronchioles.

Emphysema extent was quantified as the percentage of low attenuation area (LAA%) using a prespecified attenuation threshold of −950 HU. LAA% was calculated for the bilateral lungs and, when applicable, separately for the right and left lungs. Visible-airway dimensions were measured at the eighth-generation bronchial bifurcation in the lateral basal segment of the left lower lobe. The measured airway parameters included lumen area, wall area, wall thickness, wall area percentage, wall-to-diameter ratio, minimum inner diameter, and outer diameter. Because standard CT cannot directly resolve terminal small airways, these parameters were interpreted as visible-airway or indirect small-airway-related CT indices rather than direct measurements of terminal bronchioles.

### Bronchial-tree morphology classification

Bronchial-tree morphology was assessed using reconstructed three-dimensional airway images generated from HRCT data. CT/MRI technicians performed three-dimensional airway reconstruction on the workstation, together with multi-planar reconstruction (MPR) and minimum intensity projection (MinIP), to provide multi-angle visualization of the tracheobronchial tree. Quantitative airway measurements were obtained using Inspace software.

Based on visual assessment of the reconstructed airway tree, bronchial morphology was classified as compact or sparse type. Compact morphology was defined as a bronchial tree with relatively complete and dense visible airway branching, better peripheral branch continuity, and larger reconstructed tracheobronchial volume. Sparse morphology was defined as a bronchial tree with visibly reduced airway branching, fewer or shortened peripheral branches, discontinuous distal airway visualization, and smaller reconstructed tracheobronchial volume. Representative reconstructed images are shown in Figure 1 and Supplementary Figure 2.

**Figure 1:**
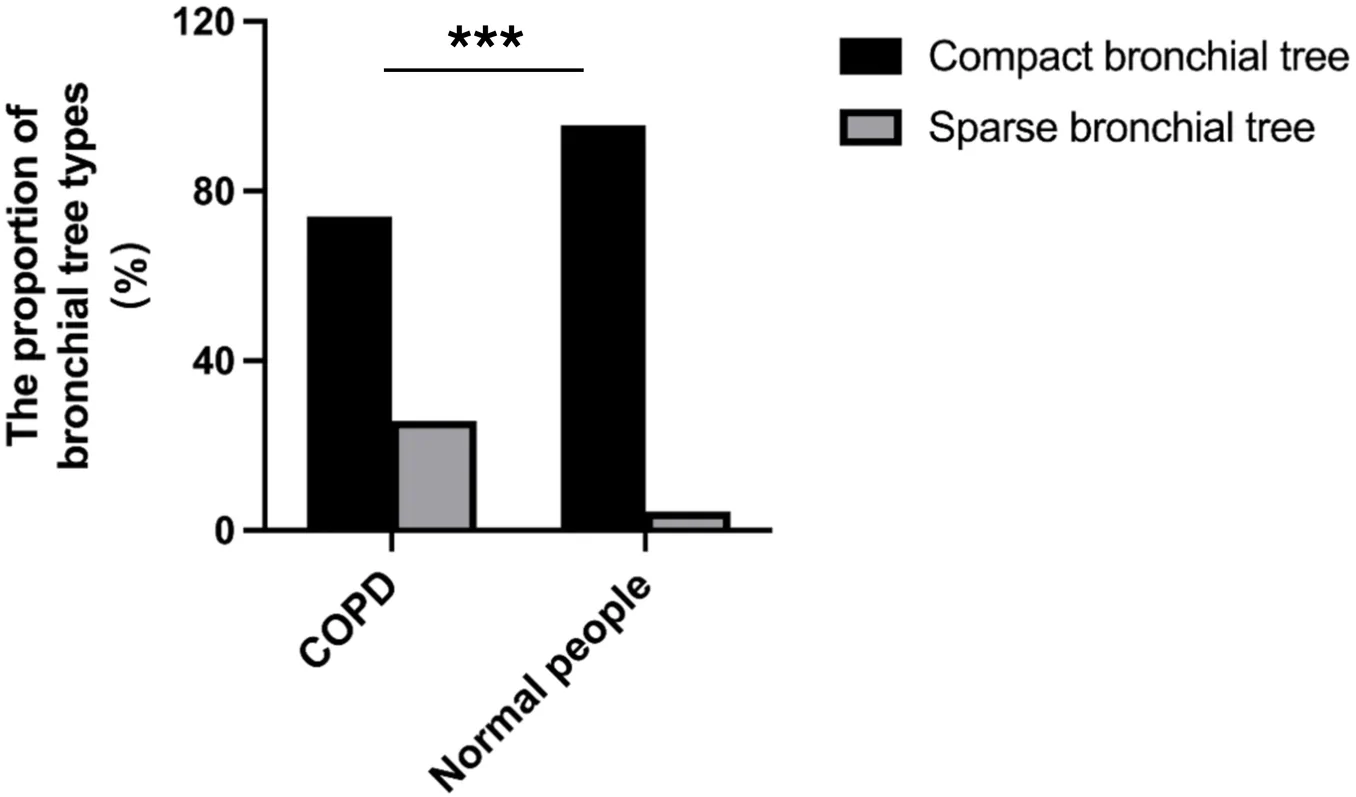
Proportions of different types of bronchial trees in COPD patients and control participants.

Because this compact/sparse classification was based on visual morphology and has not yet been fully standardized or externally validated, it was treated as a hypothesis-generating imaging feature rather than a validated imaging biomarker. Formal interobserver and intraobserver agreement testing for this visual classification was not performed in the present retrospective dataset; therefore, its reproducibility cannot be quantified from the current study.

### Statistical analysis and model-development approach

All statistical analyses were performed using SPSS version 21.0. Continuous variables were summarized as mean ± standard deviation for normally distributed data or median with interquartile range for non-normally distributed data. Categorical variables were summarized as counts and percentages. Normality was assessed before group comparisons. For comparisons between two groups, normally distributed continuous variables were analyzed using Student's t test, whereas non-normally distributed continuous variables were analyzed using the Mann–Whitney U test. For comparisons among more than two groups, one-way analysis of variance or the Kruskal–Wallis test was used, as appropriate. Categorical variables were compared using the chi-square test or Fisher's exact test when expected cell counts were small. Correlations between CT emphysema parameters and pulmonary function indices were assessed using Spearman's correlation analysis. A two-sided *P* value <0.05 was considered statistically significant.

Multivariable logistic regression was used to develop exploratory models for COPD classification and for classification of severe airflow obstruction. COPD status was defined by spirometry, and severe airflow obstruction was defined as FEV1%pred <50%, corresponding to GOLD spirometric grades 3–4. Candidate variables included clinical characteristics, emphysema parameters, visible-airway measurements, and bronchial-tree morphology. Variables associated with the outcome in univariable analysis were considered for multivariable modeling, and backward selection was used to derive the final fitted models.

Because several CT variables are anatomically or mathematically related, particularly bilateral, right-lung, and left-lung LAA%, as well as airway measurements such as lumen area, wall area, inner diameter, outer diameter, WA%, and T/D, individual regression coefficients were interpreted cautiously. Contradictory coefficient directions were considered potentially related to multicollinearity or model instability rather than definitive biological effects. A full additional analysis using correlation matrices, variance inflation factors, penalized regression, and complete model refitting was not completed in the present study and is therefore acknowledged as a limitation.

Model discrimination was assessed using receiver operating characteristic curves and area under the curve (AUC). Because the models were developed and evaluated in the same single-center cohort and no independent test set, bootstrap validation, cross-validation, or external validation was performed, the reported AUCs represent apparent performance and may be optimistic. Therefore, the models were interpreted as exploratory single-center development models rather than clinically applicable prediction tools. This approach is consistent with prediction-model reporting recommendations that emphasize internal validation, calibration, and transparent reporting (15).

## Result

### Demographic and clinical characteristics of COPD patients and control participants

Among the 229 participants, 138 had COPD and 91 were control participants. Smoking exposure was more common in the COPD group; 91.3% of COPD patients had a smoking history and 8.7% were current smokers, with a mean smoking exposure of 17.71 ± 24.4 pack-years. Given the different recruitment sources of the two groups, these differences were interpreted as baseline characteristics rather than causal estimates. The prevalence of cough, sputum production, and wheezing did not differ significantly between groups. Most COPD patients had mild dyspnea, with 70.8% classified as mMRC grades 0–1.

Pulmonary function tests revealed clear intergroup differences: COPD patients had lower FEV₁/FVC (59.51 ± 14.56 vs. 80.01 ± 5.5, *P* < 0.001) and FEV₁%pred (58.18 ± 20.02 vs. 98.05 ± 17.7, *P* < 0.001). These findings demonstrate significant disparities in pulmonary function, age, sex, and smoking exposure between groups (Table 1).

**Table 1:** Clinical characteristics of the cohort in the study (*n* = 229).

Variables	Entire population (*N* = 229)	COPD (*N* = 138)	Normal people (*N* = 91)	Statistic	*P* value
Age (years)	68.31 ± 7.11	67.09 ± 7.82	70.15 ± 5.41	−3.1329	0.0017
Gender				5.371	0.02
Male	163 (71.2)	106 (76.8)	57 (62.6)		
Female	66 (28.8)	32 (23.2)	34 (37.4)		
BMI（kg/m^2^）				2.977	0.395
Normal	108 (47.2)	67 (48.6)	41 (45.1)		
Thin	9 (3.9)	6 (4.3)	3 (3.3)		
Overweight	82 (35.8)	44 (31.9)	38 (41.8)		
Fat	30 (13.1)	21 (15.2)	9 (9.9)		
Disease duration (years)	7.56 ± 5.4	7.56 ± 5.4	=		
Smoking (packs* per year)	17.71 ± 24.4	17.71 ± 24.4	—	—	—
Smoking status				—	—
Never smoked	64 (27.9)	64 (46.4)	—		
Used to smoke	62 (27.1)	62 (44.9)	—		
Current smoker	12 (5.2)	12 (8.7)	—		
Cough				—	—
Yes	88 (38.4)	88 (63.8)	—		
No	49 (21.4)	49 (35.5)	—		
Sputum					
Yes	78 (34.1)	78 (56.5)			
No	53 (23.1)	53 (38.4)			
Shortness of breath					
Yes	111 (48.5)	111 (80.4)			
No	26 (11.4)	26 (18.8)			
Complication					
No	50 (21.8)	50 (36.2)			
One	37 (16.2)	37 (26.8)			
Two or Two plus	32 (14)	32 (23.2)			
Stabilization therapy					
Inhalation of ICS-containing drugs	78 (34.1)	78 (56.5)			
Inhalation of ICS-free drugs	38 (16.6)	38 (27.5)			
Non-inhalation of drugs	22 (9.6)	22 (15.9)			
Treatment					
ICS	2 (0.9)	2 (1.4)			
ICS + LABA	28 (12.2)	28 (20.3)			
ICS + LAMA	1 (0.4)	1 (0.7)			
LABA + LAMA	23 (10)	23 (16.7)			
ICS + LABA + LAM	41 (17.9)	41 (29.7)			
Inhalation + other oral medications	4 (1.7)	4 (2.9)			
LAMA	8 (3.5)	8 (5.8)			
LABA	7 (3.1)	7 (5.1)			
No inhaled medication	8 (3.5)	8 (5.8)			
Number of acute exacerbations 1 year prior to enrollment					
Never	100 (43.7)	100 (72.5)			
Once	8 (3.5)	8 (5.8)			
Twice	6 (2.6)	6 (4.3)			
Three times and more	1 (0.4)	1 (0.7)			
MMRC					
0	24 (10.5)	24 (17.4)			
1	74 (32.3)	74 (53.6)			
2	26 (11.4)	26 (18.8)			
3	9 (3.9)	9 (6.5)			
4	2 (0.9)	2 (1.4)			
SGRQ（in the end）	31.13 ± 17.36	31.13 ± 17.36			
FEV1/FVC	67.73 ± 15.49	59.51 ± 14.56	80.01 ± 5.5	−10.4534	＜0.001
FEV1%pre	74.16 ± 27.34	58.18 ± 20.02	98.05 ± 17.7	−11.4618	＜0.001

### Differences in CT visible-airway parameters and emphysema indicators between COPD patients and control participants

Small-airway disease and emphysema are key pathological features of COPD (16, 17). In this study, HRCT was used to assess visible-airway dimensions at a prespecified eighth-generation bronchial bifurcation in the left lower lobe (18), as illustrated in Figure 2. Because standard CT cannot directly visualize the smallest conducting airways, these CT-derived variables were interpreted as visible-airway measurements or indirect small-airway-related indices rather than direct measurements of terminal small airways.

**Figure 2:**
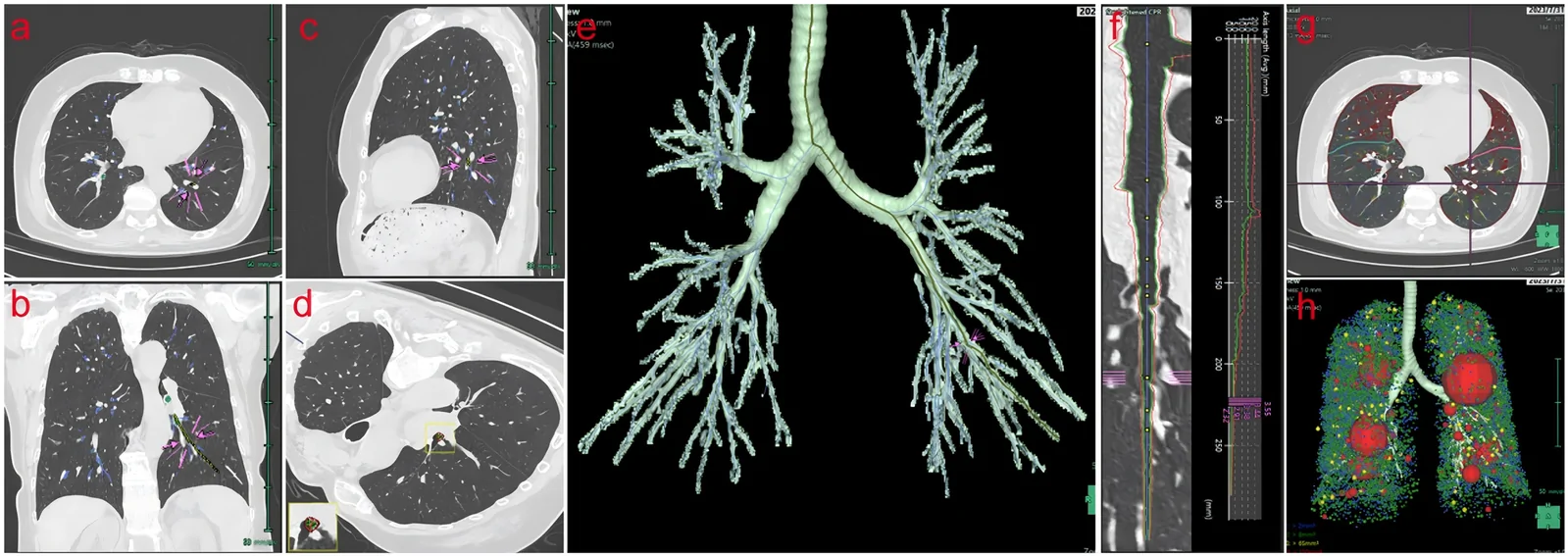
CT small airway localization and 3D model of tracheobronchial enhanced CT. **(a)** Transverse CT image of the 8th-generation bronchial bifurcation in the lateral basal segment of the left lower lobe. **(b)** Coronal CT image of the 8th-generation bronchial bifurcation in the lateral basal segment of the left lower lobe. **(c)** Sagittal CT image of the 8th-generation bronchial bifurcation in the lateral basal segment of the left lower lobe. **(d)** CT image during system measurement of the 8th-generation bronchial bifurcation in the lateral basal segment of the left lower lobe. **(e)** System-reconstructed bronchial tree. **(f)** Schematic diagram of the selected lateral basal segment of the left lower lobe bronchial generations, where each point represents a bronchial bifurcation, and the horizontal line marks the 8th-generation bifurcation. **(g)** System-generated emphysema distribution areas in this COPD patient. h. System-generated 3D simulation of emphysema in the lungs. (The area highlighted by the purple arrow indicates the 8th-generation bronchial bifurcation in the lateral basal segment of the left lower lobe).

Compared with control participants, COPD patients showed higher wall-to-diameter ratio (T/D) and wall area percentage (WA%), together with lower lumen area (LA), minimum inner diameter (Din), and outer diameter (Dout). These findings indicate airway narrowing in the COPD group. Mean wall thickness (WT) did not differ significantly between groups (*P* = 0.461), suggesting that the increase in T/D mainly reflected reduced airway lumen size rather than absolute wall thickening.

Emphysema indices—including bilateral, right, and left lung low attenuation area percentages (LAA%)—were significantly higher in COPD, reflecting structural destruction and airflow limitation. Moreover, emphysema parameters were negatively correlated with FEV₁/FVC and FEV₁%pred, indicating that increasing emphysema severity is associated with declining lung function (19) (Supplementary Table 4). These CT-based differences are summarized in Table 2.

**Table 2:** : Chest CT-based small airway and emphysema parameters in 229 subjects.

Parameters	Entire population (*N* = 229)	COPD (*N* = 138)	Normal people (*N* = 91)	Statistic	*P* value
CT-based small airway parameters					
Whole-lung 8th-generation mean wall thickness (WT), mm	0.75 ± 1.12	0.8 ± 1.46	0.68 ± 0.11	0.738	0.461
Whole-lung 8th-generation wall thickness-to-diameter ratio (T/D), WT%	32.92 ± 3.3	33.58 ± 3.42	31.96 ± 2.89	3.688	＜0.001
Whole-lung 8th-generation lumen area (LA), mm^2^	7.38 ± 3.14	6.71 ± 2.61	8.37 ± 3.58	−3.791	＜0.001
Whole-lung 8th-generation wall area (WA), mm^2^	8.39 ± 2.99	8.04 ± 2.87	8.89 ± 3.1	−2.103	0.037
Whole-lung 8th-generation wall area percentage (WA%)	54.42 ± 3.9	55.19 ± 4	53.31 ± 3.48	3.627	＜0.001
Whole-lung 8th-generation minimum inner diameter (Din), mm	2.52 ± 0.52	2.41 ± 0.46	2.69 ± 0.56	−4.057	＜0.001
Whole-lung 8th-generation minimum outer diameter (Dout), mm	3.86 ± 0.69	3.73 ± 0.63	4.05 ± 0.72	−3.53	0.001
CT-based emphysema parameters					
Total tracheobronchial volume (mL)	74.42 ± 21.19	73.91 ± 20.81	75.18 ± 21.84	−0.5207	0.6026
Both lungs，LAA%	25.25 ± 11.12	29.4 ± 10.88	19.06 ± 8.25	−7.1323	＜0.001
Right lung，LAA%	25.26 ± 11.33	29.44 ± 11.27	19.01 ± 8.13	−7.0735	＜0.001
Left lung，LAA%	25.23 ± 11.2	29.36 ± 10.91	19.05 ± 8.48	−6.9982	＜0.001

### Association of bronchial-tree morphology with COPD status

#### Distribution of compact and sparse bronchial-tree morphology in COPD patients and control participants

Bronchial-tree morphology was assessed using reconstructed three-dimensional airway images and categorized as compact or sparse type for exploratory analysis. As shown in Figure 1, compact-type morphology was the predominant pattern in both COPD patients and control participants. However, the proportion of compact-type bronchial trees was higher among control participants, whereas sparse-type morphology was more frequently observed among COPD patients (*χ*^2^ = 18.4207, *P* < 0.0001).

These findings suggest that sparse bronchial-tree morphology was associated with COPD status in this cohor (20, 21). However, because the compact/sparse classification has not yet been fully standardized or externally validated, this result should be interpreted as hypothesis-generating rather than as evidence of a validated imaging biomarker or a definitive morphological transition during COPD progression.

Differences in small airway parameters and emphysema indices between compact and sparse bronchial types are summarized in Supplementary Supplementary Tables S1–S2.

#### Multivariable logistic regression model for COPD classification

COPD status was used as the dependent variable. Variables that were significant in univariable analysis were considered for multivariable logistic regression, including age, bronchial-tree morphology, wall-to-diameter ratio (T/D), lumen area (LA), wall area (WA), minimum inner diameter (Din), outer diameter (Dout) of the eighth-generation airway, and bilateral, right-lung, and left-lung LAA%. Backward stepwise selection was used to derive the final fitted model.

As shown in Table 3, the variables retained in the multivariable model were age, sparse bronchial-tree morphology, lumen area, wall area, and LAA% parameters. Age was inversely associated with COPD status in the fitted model (OR = 0.922, 95% CI: 0.862–0.978). Sparse bronchial-tree morphology was associated with higher odds of COPD (OR = 28.596, 95% CI: 6.707–149.774), although the wide confidence interval indicates statistical uncertainty. Lumen area was inversely associated with COPD status (OR = 0.509, 95% CI: 0.370–0.670), whereas wall area was positively associated with COPD status (OR = 1.451, 95% CI: 1.140–1.907).

**Table 3:** Multivariable logistic regression model combining clinical characteristics and HRCT emphysema/visible-airway parameters for COPD classification.

Parameters	Estimate	Std.Error	z.value	Pr(>|z|)	OR	95%CI
（intercept）	3.113	2.349	1.325	0.185	22.494	(0.29，2832.863)
Age（years）	−0.081	0.032	−2.538	0.011	0.922	(0.862，0.978)
Bronchial Tree Classification						
Compact	ref	ref	ref	ref	ref	ref
Sparse	3.353	0.786	4.269	＜0.001	28.596	(6.707，149.774)
Whole-lung 8th-generation lumen area (LA), mm^2^	−0.676	0.151	−4.478	＜0.001	0.509	(0.37，0.67)
Whole-lung 8th-generation wall area (WA), mm^2^	0.372	0.13	2.854	0.004	1.451	(1.14，1.907)
Bothlungs LAA%	−5.486	1.963	−2.795	0.005	0.004	(0，0.168)
Rightlung LAA%	2.966	1.075	2.758	0.006	19.413	(2.583，176.059)
Leftlung LAA%	2.706	0.901	3.002	0.003	14.965	(2.714，94.669)

Bilateral LAA% showed an inverse coefficient in the fitted model (OR = 0.004, 95% CI: 0–0.168), whereas right-lung LAA% (OR = 19.413, 95% CI: 2.583–176.059) and left-lung LAA% (OR = 14.965, 95% CI: 2.714–94.669) showed positive coefficients. Because bilateral, right-lung, and left-lung LAA% are anatomically and mathematically related, these inconsistent coefficient directions may reflect multicollinearity or model instability rather than biologically meaningful effects. Therefore, the individual LAA% coefficients should be interpreted cautiously.

Overall, the fitted model suggests that sparse bronchial-tree morphology, smaller lumen area, greater wall area, and emphysema-related CT parameters were associated with spirometry-defined COPD in this cohort. These associations should not be interpreted causally.

The ROC curve in Figure 3 shows the apparent discrimination of the single-center COPD classification development model, with an AUC of 0.906. At a cutoff value of 0.691, the sensitivity was 96.7% and the specificity was 76.3%. Because the model was developed and evaluated in the same cohort without an independent test set, bootstrap validation, cross-validation, or external validation, this performance estimate should be interpreted as apparent and potentially optimistic.

**Figure 3:**
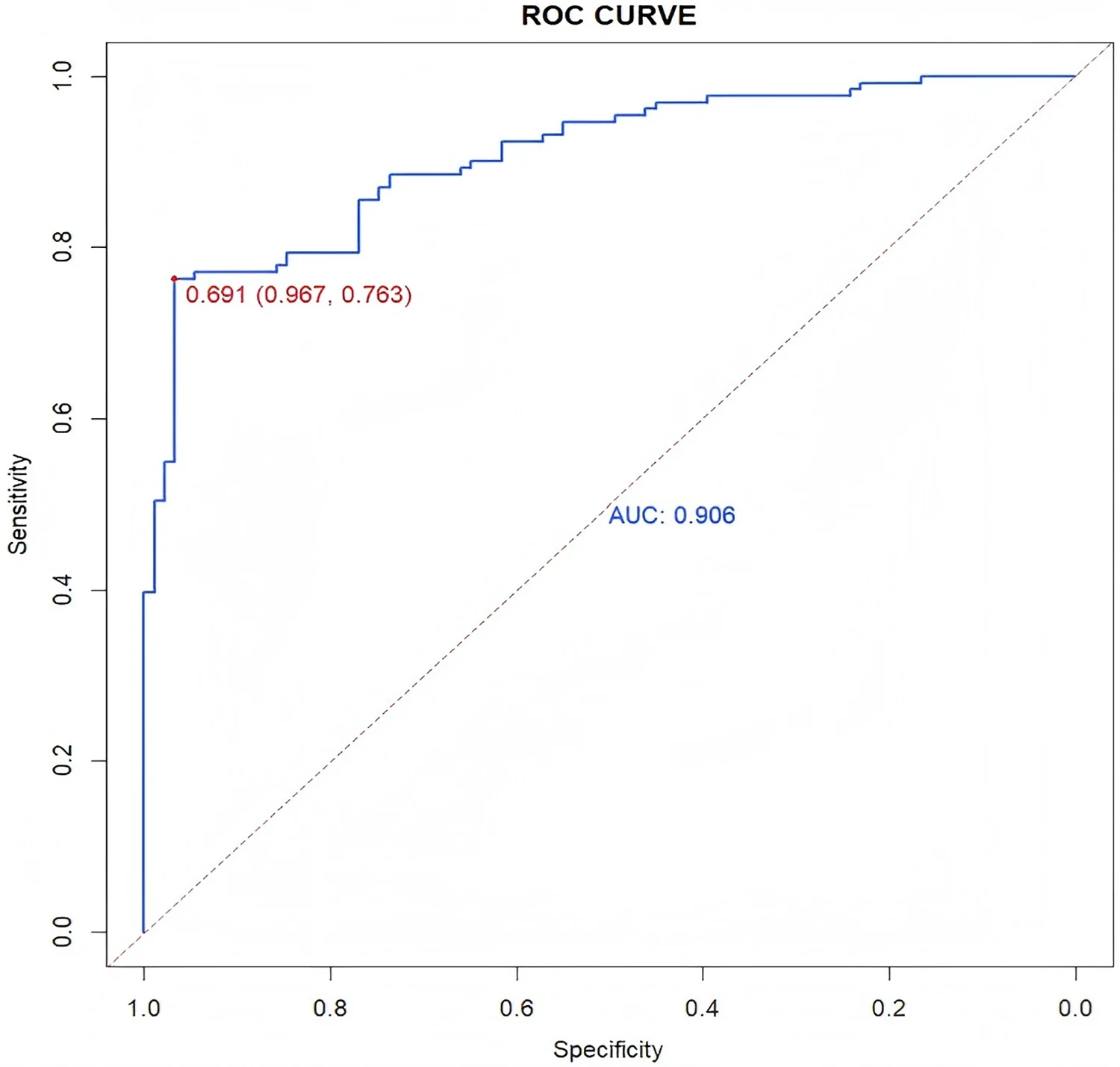
ROC curve for the singlE,center COPD classification development model.

Demographic and Clinical Characteristics of Mild-to-Moderate COPD and GOLD Spirometric Grades 3–4 COPD

To explore factors associated with spirometric severity, COPD patients with available FEV1%pred data were divided into a mild-to-moderate group and a severe airflow obstruction group. Mild-to-moderate COPD was defined as FEV1%pred ≥50%, whereas severe airflow obstruction was defined as FEV1%pred <50%, corresponding to GOLD spirometric grades 3–4. After excluding two COPD participants without complete FEV1%pred data, 136 COPD patients were included in this severity analysis.

Compared with the mild-to-moderate group, the severe airflow obstruction group had a higher proportion of male patients (90.2% vs. 68.2%). No significant differences were observed in age or BMI between the two groups.

Severe COPD patients showed longer disease duration, greater smoking pack-years, and a higher proportion of former smokers (58.8%). Lung function indices (FEV₁/FVC and FEV₁%pred) were markedly reduced, reflecting greater impairment. The mMRC score and SGRQ score were significantly higher in the severe group (36.5 ± 15.78 vs. 26.2 ± 17.37, *P* = 0.014), indicating worse symptoms and quality of life.

No significant difference was found in the number of exacerbations over the previous year. Although inhaled corticosteroid (ICS) use was more common in the severe group (62.7% vs. 51.8%), treatment strategies were otherwise similar.

As summarized in Supplementary Table 3, severe COPD patients were predominantly male, had longer disease duration, heavier smoking histories, lower lung function, poorer quality of life, more comorbidities, and higher mMRC scores, confirming their greater clinical and functional burden.

Differences in CT Emphysema Parameters and Visible-Airway Parameters Between Mild-to-Moderate COPD and GOLD Spirometric Grades 3–4 COPD

As shown in Table 4, bilateral lung LAA% was significantly higher in patients with GOLD spirometric grades 3–4 than in those with mild-to-moderate COPD (34.9 ± 10.25% vs. 26.5 ± 9.9%), indicating more extensive emphysema in participants with lower FEV1%pred. Similar differences were observed for right-lung LAA% (35.3 ± 11.04% vs. 26.3 ± 9.89%) and left-lung LAA% (34.4 ± 10.18% vs. 26.7 ± 10.21%). These findings support an association between greater emphysema extent and more severe spirometric airflow obstruction.

**Table 4:** Differences in CT visible-airway parameters and CT emphysema parameters across GOLD spirometric severity levels.

Parameters	COPD		Statistic	*P* value
	Mild to moderate (*N* = 85)	severe (*N* = 51)		
Total tracheobronchial volume (mL)	73.1 ± 21.6	76.5 ± 19.07	−0.282	0.778
Both lungs，LAA%	26.5 ± 9.9	34.9 ± 10.25	−4.66	＜0.001
Right lung，LAA%	26.3 ± 9.89	35.3 ± 11.04	−4.775	＜0.001
Left lung，LAA%	26.7 ± 10.21	34.4 ± 10.18	−4.219	＜0.001
Whole-lung 8th-generation mean wall thickness (WT), mm	0.9 ± 1.85	0.7 ± 0.11	−0.022	0.983
Whole-lung 8th-generation wall thickness-to-diameter ratio (T/D), WT%	33.3 ± 3.25	34 ± 3.68	−1.721	0.085
Whole-lung 8th-generation lumen area (LA), mm^2^	6.8 ± 2.61	6.7 ± 2.65	−0.335	0.737
Whole-lung 8th-generation wall area (WA), mm^2^	8.1 ± 3.01	8 ± 2.69	−0.082	0.935
Whole-lung 8th-generation wall area percentage (WA%)	54.8 ± 3.97	55.7 ± 4.02	−1.709	0.088
Whole-lung 8th-generation minimum inner diameter (Din), mm	2.4 ± 0.47	2.4 ± 0.46	−0.543	0.587
Whole-lung 8th-generation minimum outer diameter (Dout), mm	3.7 ± 0.63	3.7 ± 0.64	−0.217	0.828

In contrast, visible-airway CT parameters did not differ significantly between the two groups, suggesting that emphysema extent, rather than visible-airway measurements, was more closely associated with spirometric severity in this cohort.

As illustrated in Figure 4, the distribution of compact and sparse bronchial-tree morphology was broadly comparable across GOLD spirometric severity groups. Sparse and compact morphologies were observed across different severity grades, without a clear monotonic trend with increasing spirometric severity. This suggests that bronchial-tree morphology was not clearly associated with GOLD spirometric grade in the present cohort.

**Figure 4:**
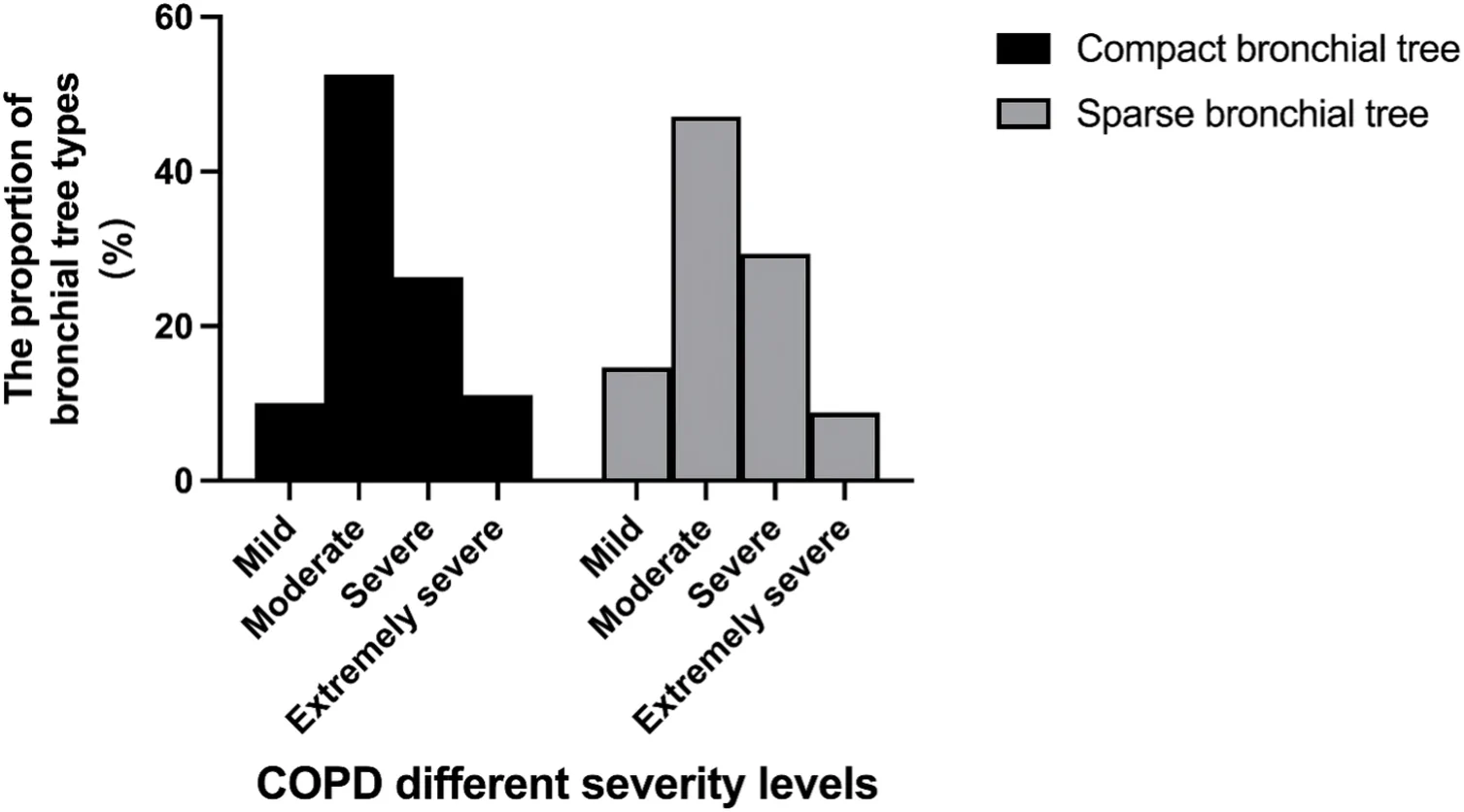
Distribution of sparsE–type and compacT,type bronchial morphologies across GOLD spirometric severity levels.

#### Multivariable Logistic Regression Model for Classifying Severe Airflow Obstruction

Severe airflow obstruction, defined as FEV1%pred <50% versus ≥50%, was used as the dependent variable. Variables that were significant in univariable analysis were considered for multivariable logistic regression, including sex, smoking status, comorbidity category, disease duration, smoking pack-years, bilateral LAA%, right-lung LAA%, and left-lung LAA%. Backward stepwise selection was used to derive the final fitted model.

As shown in Table 5, disease duration, smoking pack-years, bilateral LAA%, and comorbidity category were retained in the final model. Disease duration (OR = 1.113, 95% CI: 1.011–1.242), smoking pack-years (OR = 1.028, 95% CI: 1.006–1.052), and bilateral LAA% (OR = 1.109, 95% CI: 1.046–1.189) were positively associated with severe airflow obstruction. One comorbidity category showed an inverse association with severe airflow obstruction (OR = 0.075, 95% CI: 0.013–0.320), whereas two or more comorbidities were not significantly associated with severe airflow obstruction (OR = 1.299, 95% CI: 0.366–4.822; *P* = 0.688).

**Table 5:** Multivariable logistic regression model for classifying severe airflow obstruction.

Parameters	Estimate	Std.Error	z.value	Pr(>|z|)	OR	95%CI
(Intercept)	−4.583	1.216	−3.767	＜0.001	0.01	(0.001，0.087)
Complication						
No	ref	ref	ref	ref	ref	ref
One	−2.587	0.809	−3.198	0.001	0.075	(0.013，0.32)
Two or Two plus	0.261	0.65	0.402	0.688	1.299	(0.366，4.822)
Disease duration (years)	0.107	0.051	2.076	0.038	1.113	(1.011，1.242)
Smoking (packs* per year)	0.027	0.011	2.408	0.016	1.028	(1.006，1.052)
Bothlungs LAA%	0.103	0.032	3.197	0.001	1.109	(1.046，1.189)

These findings suggest that longer disease duration, greater smoking exposure, and greater bilateral emphysema extent were associated with severe spirometric airflow obstruction in this cohort. The inverse association observed for one comorbidity should be interpreted cautiously because it may reflect subgroup size, reference-category coding, residual confounding, or model instability rather than a protective effect.

The ROC curve in Figure 5 shows the apparent discrimination of the single-center development model for classifying severe airflow obstruction. The model had an apparent AUC of 0.859. At a cutoff value of 0.350, the sensitivity was 85.3% and the specificity was 75.0%. Because this model was developed and evaluated in the same cohort, these performance estimates should be interpreted as apparent and potentially optimistic until validated in independent cohorts.

**Figure 5:**
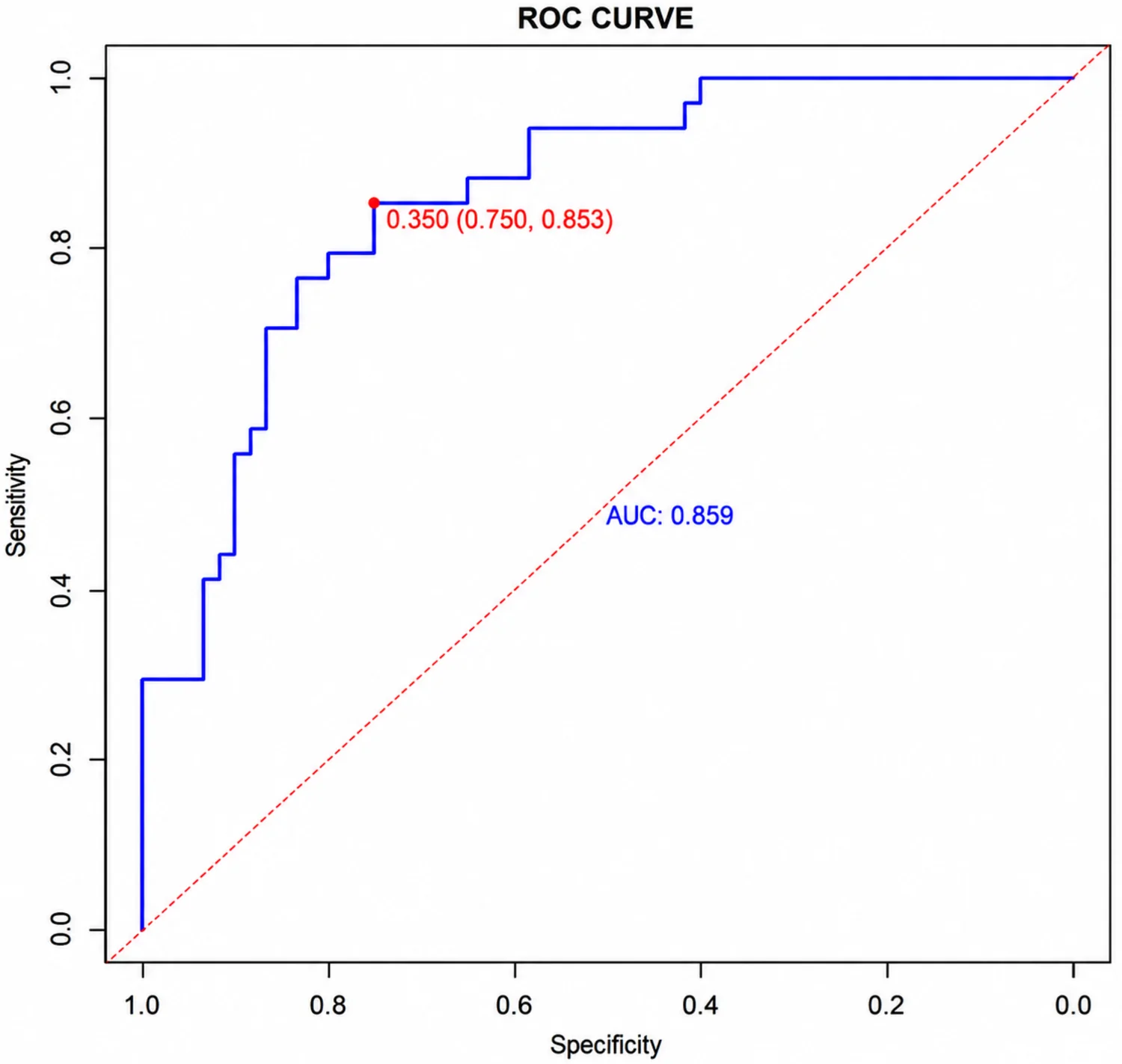
ROC curve for the single-center severe airflow obstruction development model.

## Discussion

This study investigated the structural characteristics of COPD using HRCT-derived emphysema burden, visible-airway measurements, and bronchial-tree morphology, and further developed multivariable models integrating imaging and clinical variables. The main findings were that CT-derived emphysema extent and airway morphological parameters were associated with spirometry-defined COPD, and that emphysema extent was associated with GOLD spirometric grades 3–4. These observations are broadly consistent with previous quantitative CT studies showing associations between emphysema, airway dimensions, and airflow limitation. Therefore, the primary contribution of this study should be interpreted as exploratory model development and bronchial-morphology assessment, rather than the discovery of entirely new CT biomarkers.

Spirometry remains the diagnostic standard for COPD and is cheaper, more widely available, and free of radiation exposure. However, pulmonary function tests alone may not fully characterize the structural heterogeneity of COPD, especially when early pathological changes, heterogeneous emphysema distribution, or airway remodeling are present. In addition, spirometric performance may be affected by patient cooperation, and age-related decline in lung function may complicate interpretation in older adults. Thus, HRCT should not be viewed as a replacement for spirometry, but rather as a complementary phenotyping tool when CT is already clinically indicated (14, 22). In the present study, the CT-based models predicted spirometric labels, including COPD and severe airflow obstruction, rather than independent clinical outcomes such as mortality, exacerbations, treatment response, or long-term prognosis (23–25). Future studies should therefore determine whether CT features provide incremental prognostic or therapeutic value beyond PFT and conventional clinical variables.

From a mechanistic perspective, COPD progression involves both small airway disease and emphysema, which frequently coexist and interact (26). Airway inflammation and remodeling may lead to airway narrowing, while emphysema reflects alveolar destruction and loss of elastic recoil (27). In this study, HRCT-derived airway measurements showed smaller lumen area and higher wall-related ratios in COPD, while emphysema extent was negatively associated with FEV₁/FVC and FEV₁%pred, consistent with previous quantitative CT studies (28, 29). These findings support the complementary structural information provided by CT. Nevertheless, standard CT cannot directly resolve terminal small airways; therefore, the airway variables should be interpreted as visible-airway measurements or indirect small-airway-related indices rather than direct measurements of terminal bronchioles.

The compact/sparse bronchial-tree morphology remains the most distinctive exploratory feature of this study. Sparse morphology was more frequent in COPD and retained a strong association in the fitted multivariable model (OR = 28.596), but the very wide 95% CI (6.707–149.774) indicates substantial imprecision; the magnitude of the OR should therefore not be interpreted as a stable effect size. Several factors may contribute to this estimate. Compact morphology predominated overall, so the relatively low frequency of the sparse category may have contributed to sparse-data effects. In addition, the binary visual classification was modeled together with multiple structural CT variables, making residual confounding, correlated predictors, and model instability plausible. Apparent distal branch sparsity may also be influenced by inspiratory level, reconstruction or segmentation quality, and peripheral-branch visibility, rather than reflecting airway loss alone. Formal interobserver and intraobserver agreement testing was not performed in the present retrospective dataset, so reproducibility cannot yet be quantified. Accordingly, this finding should remain hypothesis-generating until the classification is operationally standardized and reproducibility is demonstrated.

The regression coefficients should be interpreted as cohort-specific conditional associations rather than epidemiologic effects. In particular, age was inversely associated with COPD status after multivariable adjustment (OR = 0.922), which should not be interpreted as indicating that older age is protective or as contradicting the established increase in COPD prevalence with aging (30). Cases and controls were recruited from different source populations, with baseline differences in age and other characteristics; moreover, the fitted model simultaneously included several structural CT variables and used backward selection. Under these conditions, confounding, suppression effects, correlated predictors, and model-selection instability can change the direction or magnitude of an adjusted coefficient. The inverse age estimate is therefore more plausibly regarded as a cohort- and model-specific finding than as a biological age effect. Similar caution applies to the contradictory bilateral and unilateral LAA% coefficients, which may reflect the strong mathematical dependence among these measures. The reported AUCs are also apparent development-cohort estimates and require internal validation, calibration assessment, and external validation before clinical use.

Recruitment design remains an important source of potential bias. COPD patients were drawn from an outpatient clinic and controls from a health examination center, which may introduce selection and spectrum bias and may contribute to the apparent discrimination of the COPD model. Matching or sensitivity analyses accounting for age, sex, and smoking exposure were not performed and should be considered in future work. In the severity analysis, FEV₁%pred <50% represents severe spirometric airflow obstruction rather than multidimensional clinical severity. The inverse association for one comorbidity category should likewise not be interpreted as protective, because subgroup size, reference-category coding, residual confounding, selection bias, and model instability remain plausible explanations.

This study has several limitations. It is a single-center retrospective model-development study with a moderate sample size, different recruitment sources for cases and controls, and no internal or external validation, which limits generalizability and may produce optimistic performance estimates. The early-stage COPD analysis was not predefined sufficiently for strong inference (31). The CT airway measurements represent visible-airway or indirect surrogates rather than direct terminal-bronchiolar measurements, and the compact/sparse morphology classification lacks formal interobserver and intraobserver reproducibility data. Potential multicollinearity among emphysema and airway variables may also have affected coefficient stability. Future studies should use representative prospective cohorts, standardized morphology criteria with reader-reproducibility testing, more parsimonious or penalized modeling, and prespecified internal validation, calibration, and external validation (32, 33).

In summary, quantitative CT characterized emphysema burden, visible-airway remodeling, and bronchial-tree morphology associated with spirometry-defined COPD in this cohort. Sparse bronchial morphology is an exploratory signal rather than a validated biomarker, and the regression models should be regarded as single-center development models with apparent performance. HRCT may complement spirometry for structural phenotyping when clinically indicated, but further validation is required before clinical application.

## Conclusion

In summary, CT-detected emphysema and CT-derived visible-airway parameters were associated with spirometry-defined COPD in this single-center cohort. Emphysema extent, particularly bilateral LAA%, was associated with severe airflow obstruction (FEV1%pred < 50%), whereas visible-airway parameters did not differ significantly between spirometric severity groups. The two logistic-regression models should be considered development models with apparent performance only. Sparse bronchial morphology is a potentially informative but exploratory feature that requires reproducible definition, quality control, and external validation. CT may complement, but not replace, spirometry and clinical assessment.

## Data Availability

The raw data supporting the conclusions of this article will be made available by the authors, without undue reservation.
