# Supplementary material for: Quantitative CT features for COPD classification and GOLD spirometric severity assessment: a single-center model-development study
Source: Front Med (Lausanne). 2026 Oct 1;13:1900490. doi: 10.3389/fmed.2026.1900490 (PMC13669100; doi:10.3389/fmed.2026.1900490)
Supplement: Supplementary file 1 [file Datasheet1.docx]

**The Supplementary Methods**

**Data Collection：**

General Information: The hospital's electronic medical record system was accessed to retrieve and collect patients' data at admission, including age, sex, body mass index (BMI), comorbidities (diabetes, hypertension, hyperlipidemia), smoking history, smoking pack-years (number of cigarette packs smoked per day × number of years smoked), disease status [COPD duration, symptoms (cough, sputum production, wheezing), and the number of acute exacerbations in the past year], treatment methods, mMRC score (total score of 4, with higher scores indicating more severe dyspnea), and St. George's Respiratory Questionnaire (SGRQ).

Laboratory Tests: Pulmonary function tests were performed on all patients and healthy individuals enrolled in the study using the MasterScreen pulmonary function instrument from Jaeger, Germany. The measured parameters included forced expiratory volume in one second (FEV_1_) and forced vital capacity (FVC).

HRCT and CT Tracheal Tree 3D Reconstruction: High-Resolution CT (HRCT) Examination: Scans were performed using a Siemens Somatom Definition AS 32-slice, 64-layer CT scanner. Patients were positioned supine with arms raised above the head. They were instructed to inhale deeply and hold their breath during the scan. The system reconstructed the scanning range with a 1 mm slice thickness to achieve HRCT imaging. Technicians then performed sagittal reconstructions focusing on lesion areas. HRCT scans were repeated every three months for six months, during which patients' smoking cessation status was recorded.Images were reviewed using a PACS workstation. Two experienced radiologists, blinded to clinical data, utilized Inspace software to calculate emphysema areas. The boundary between normal lung parenchyma and low attenuation areas (LAA) was defined as <−950 HU, and the proportion of emphysematous tissue in the entire lung (low attenuation area percentage, LAA%) was calculated. Inspace software was also used to measure parameters of the 8th generation of small airways across the entire lung, including wall thickness (WT), wall thickness percentage (WT% = wall thickness/external diameter, T/D), wall area (WA), lumen area (LA), lumen area percentage (WA%), minimum internal diameter (Din), and minimum external diameter (Dout).CT Tracheal Tree 3D Reconstruction: CT/MRI technicians utilized the workstation to perform 3D airway reconstruction, multi-planar reconstruction (MPR), and minimum intensity projection (MinIP) techniques, providing multi-angled views of the tracheal morphology.

**Statistical Analysis**

Statistical analysis was performed using SPSS 21.0 software. Measurement data were first tested for normality, and data conforming to a normal distribution were expressed as mean ± standard deviation (xˉ±s). One-way analysis of variance (ANOVA) and independent samples t-test were used for group comparisons. The correlation between LAA% and pulmonary function parameters was analyzed using Spearman's correlation coefficient (rr). Categorical data were compared between groups using the chi-square (χ^2^) test. A P<0.05P<0.05 was considered statistically significant.

Supplementary Figure 1. Selection of Study Subjects and Research Process

Supplementary Figure 2. Selection of Study Subjects and Research Process

**II.Differences in CT Small Airway Parameters and Emphysema Indicators Among Individuals with Different Bronchial Morphologies in the COPD and Healthy Groups**.（STable 1）

a. Differences in the COPD Group:

In COPD patients, those with compact bronchial trees had significantly larger total tracheobronchial volumes compared to those with sparse bronchial trees. Additionally, the LAA% for both lungs, the right lung, and the left lung was significantly higher in compact-type patients. The mean airway wall thickness (WT) was also significantly greater in compact-type patients. The wall-to-diameter ratio (T/D) was lower in the compact type but showed a significant difference (P = 0.015). Compact-type patients had significantly larger lumen areas (LA) and wall areas (WA) compared to sparse-type patients. The wall area percentage (WA%) was lower in the compact type but showed a significant difference (P = 0.044). The minimum inner diameter (Din) and outer diameter (Dout) were both significantly larger in compact-type patients, indicating that compact bronchial trees possess greater airway volume, larger diameters, and a thicker airway wall.

b. Differences in the Healthy Population:

Similar to the COPD group, individuals with compact bronchial trees in the healthy population had significantly larger total tracheobronchial volumes compared to those with sparse bronchial trees. The emphysema indicator (LAA%) was significantly higher in compact-type bronchial trees. The airway wall thickness (WT) in compact-type bronchial trees was also significantly greater than in sparse-type individuals. Compact bronchial trees had significantly larger lumen areas (LA) and wall areas (WA), but no significant difference was observed in WA%. The minimum inner diameter (Din) and outer diameter (Dout) were significantly larger in compact-type bronchial trees. However, the wall-to-diameter ratio (T/D) was higher in the compact type, but the difference was not significant (P = 0.169).

We observed significant differences in CT small airway and emphysema parameters between the two bronchial tree types in COPD patients. Compared to sparse bronchial trees, compact bronchial trees exhibited larger tracheobronchial volumes, higher emphysema indicators (LAA%), thicker airway walls, larger lumen and wall areas, and wider inner and outer diameters. A similar pattern was observed in the healthy population, though the wall-to-diameter ratio (T/D) and wall area percentage (WA%) showed no significant differences between the two types of bronchial trees.

**III．Demographic Data and Clinical Characteristics of COPD Patients with Sparse-Type and Compact-Type Bronchial Morphologies**（S Table 2）

Overall, there were no significant differences between the two groups of COPD patients, classified by different bronchial types, in terms of sex, age, BMI, disease duration, smoking status, symptoms, comorbidities, treatment regimens, quality of life scores, or lung function. Although cough symptoms (P = 0.06) and treatment regimens (P = 0.077) were close to the threshold of statistical significance, they did not reach the level of statistical significance. This may indicate that the baseline characteristics and clinical presentations of the two groups are similar, indirectly suggesting that the two different bronchial types in COPD patients have no impact on disease severity ,clinical symptoms, signs, and pulmonary function.

**Supplementary Table 1 Differences in CT Small Airway Parameters and Emphysema Indicators Among Individuals with Different Bronchial Morphologies in the COPD and Healthy Groups**

|  | COPD（N=135） | | | | Normal People (N=91) | | | |
| --- | --- | --- | --- | --- | --- | --- | --- | --- |
|  | Compact bronchial tree（N=100） | Sparse bronchial tree（N=35） | Statistic | P value | Compact bronchial tree（N=87） | Sparse bronchial tree（N=4） | Statistic | P value |
| Total tracheobronchial volume (ml) | 78.2±20.02 | 60.47±17.04 | -4.515 | ＜0.001 | 76.68±20.94 | 42.7±16.73 | -2.71 | 0.007 |
| Both lungs，LAA% | 30.98±9.77 | 24.96±12.82 | -2.664 | 0.008 | 19.48±8.09 | 10±7.2 | -1.994 | 0.046 |
| Right lung，LAA% | 30.77±10.05 | 25.73±13.78 | -2.217 | 0.027 | 19.42±7.96 | 10.13±7.5 | -1.985 | 0.047 |
| Left lung，LAA% | 31.22±9.76 | 24.1±12.53 | -3.251 | 0.001 | 19.48±8.32 | 9.73±7.16 | -1.975 | 0.048 |
| Whole-lung 8th-generation mean wall thickness (WT), mm | 0.85±1.68 | 0.62±0.11 | -2.808 | 0.005 | 0.69±0.11 | 0.55±0.12 | -2.188 | 0.029 |
| Whole-lung 8th-generation wall thickness-to-diameter ratio (T/D), WT% | 33.25±3.16 | 34.85±3.8 | -2.434 | 0.015 | 31.82±2.69 | 35.06±5.42 | -1.376 | 0.169 |
| Whole-lung 8th-generation lumen area (LA), mm² | 7.06±2.44 | 5.3±2.24 | -3.815 | ＜0.001 | 8.56±3.52 | 4.26±2.44 | -2.401 | 0.016 |
| Whole-lung 8th-generation wall area (WA), mm² | 8.47±2.87 | 6.57±2.35 | -3.707 | ＜0.001 | 9.07±3.03 | 5.01±2.14 | -2.498 | 0.013 |
| Whole-lung 8th-generation wall area percentage (WA%) | 54.97±3.58 | 56.22±4.82 | -2.013 | 0.044 | 53.18±3.39 | 56.19±4.83 | -1.317 | 0.188 |
| Whole-lung 8th-generation minimum inner diameter (Din), mm | 2.49±0.44 | 2.1±0.37 | -4.35 | ＜0.001 | 2.73±0.53 | 1.88±0.51 | -2.479 | 0.013 |
| Whole-lung 8th-generation minimum outer diameter (Dout), mm | 3.84±0.62 | 3.35±0.5 | -3.943 | ＜0.001 | 4.1±0.69 | 3.01±0.66 | -2.498 | 0.012 |

**Supplementary Table 2 Demographic Data and Clinical Characteristics of COPD Patients with Sparse-Type and Compact-Type Bronchial Morphologies**

|  | COPD | | Statistic | P value |
| --- | --- | --- | --- | --- |
|  | Compact bronchial tree（N=100） | Sparse bronchial tree（N=35） |  |  |
| Gender |  |  | 0.202 | 0.653 |
| Male | 78(78) | 26(74.3) |  |  |
| Female | 22(22) | 9(25.7) |  |  |
| Age (years) | 67.35±7.84 | 66.89±7.86 | -0.065 | 0.948 |
| BMI（kg/m^2^） |  |  | 1.624 | 0.666 |
| Normal | 49(49) | 17(48.6) |  |  |
| Thin | 5(5) | 1(2.9) |  |  |
| Overweight | 34(34) | 10(28.6) |  |  |
| Fat | 12(12) | 7(20) |  |  |
| Disease duration (years) | 7.68±5.68 | 7.31±4.85 | -0.036 | 0.971 |
| Smoking (packs* per year) | 17.19±24.32 | 19.46±25.67 | -0.241 | 0.809 |
| Smoking status |  |  | 1.563 | 0.488 |
| Never smoked | 46(46) | 17(48.6) |  |  |
| Used to smoke | 44(44) | 17(48.6) |  |  |
| Current smoker | 10(10) | 1(2.9) |  |  |
| Cough |  |  | 3.544 | 0.06 |
| Yes | 68(68) | 17(48.6) |  |  |
| No | 32(32) | 17(48.6) |  |  |
| Sputum |  |  | 0.083 | 0.774 |
| Yes | 56(56) | 21(60) |  |  |
| No | 39(39) | 13(37.1) |  |  |
| Shortness of breath |  |  | 1.354 | 0.245 |
| Yes | 79(79) | 31(88.6) |  |  |
| No | 20(20) | 4(11.4) |  |  |
| Complication |  |  | 0.258 | 0.879 |
| No | 33(33) | 14(40) |  |  |
| One | 26(26) | 11(31.4) |  |  |
| Two or Two plus | 24(24) | 8(22.9) |  |  |
| Stabilization therapy |  |  | 0.847 | 0.655 |
| Inhalation of ICS-containing drugs | 55(55) | 22(62.9) |  |  |
| Inhalation of ICS-free drugs | 28(28) | 9(25.7) |  |  |
| Non-inhalation of drugs | 17(17) | 4(11.4) |  |  |
| Treatment |  |  | 12.809 | 0.077 |
| ICS |  | 2(5.7) |  |  |
| ICS+LABA | 18(18) | 10(28.6) |  |  |
| ICS+LAMA |  |  |  |  |
| LABA+LAMA | 17(17) | 5(14.3) |  |  |
| ICS+LABA+LAM | 31(31) | 10(28.6) |  |  |
| Inhalation + other oral medications | 4(4) |  |  |  |
| LAMA | 4(4) | 4(11.4) |  |  |
| LABA | 5(5) | 2(5.7) |  |  |
| No inhaled medication | 7(7) |  |  |  |
| Number of acute exacerbations 1 year prior to enrollment |  |  | 3.824 | 0.261 |
| Never | 69(69) | 29(82.9) |  |  |
| Once | 8(8) |  |  |  |
| Twice | 4(4) | 2(5.7) |  |  |
| Three times and more | 1(1) |  |  |  |
| mMRC |  |  | 5.837 | 0.19 |
| 0 | 15(15) | 9(25.7) |  |  |
| 1 | 57(57) | 14(40) |  |  |
| 2 | 19(19) | 7(20) |  |  |
| 3 | 5(5) | 4(11.4) |  |  |
| 4 | 1(1) | 1(2.9) |  |  |
| SGRQ（in the end） | 32.29±18.11 | 28.88±15.97 | -0.624 | 0.532 |
| FEV1/FVC | 59.99±14.88 | 57.43±12.64 | -0.872 | 0.383 |
| FEV1%pred | 58.14±20.24 | 58.37±19.25 | -0.15 | 0.881 |

**Supplementary Table 3 Clinical Characteristics Analysis of COPD Patients with Different Severity Levels**

|  | COPD | | Statistic | P value |
| --- | --- | --- | --- | --- |
|  | Mild to moderate  (N=85) | Severe  (N=51) |  |  |
| Age (years) | 66.7±8.85 | 67.8±5.91 | -0.74 | 0.459 |
| Gender |  |  | 8.544 | 0.003 |
| Male | 58(68.2) | 46(90.2) |  |  |
| Female | 27(31.8) | 5(9.8) |  |  |
| BMI（kg/m^2^） |  |  | 1.304 | 0.741 |
| Normal | 40(47.1) | 26(51) |  |  |
| Thin | 3(3.5) | 3(5.9) |  |  |
| Overweight | 27(31.8) | 16(31.4) |  |  |
| Fat | 15(17.6) | 6(11.8) |  |  |
| Disease duration (years) | 6.6±4.63 | 9.2±6.27 | -2.193 | 0.028 |
| Smoking (packs* per year) | 13.2±21.34 | 25.5±27.6 | -3.079 | 0.002 |
| Smoking status |  |  | 7.645 | 0.022 |
| Never smoked | 47(55.3) | 16(31.4) |  |  |
| Used to smoke | 31(36.5) | 30(58.8) |  |  |
| Current smoker | 7(8.2) | 5(9.8) |  |  |
| Cough |  |  | 1.117 | 0.29 |
| Yes | 57(67.1) | 29(56.9) |  |  |
| No | 28(32.9) | 21(41.2) |  |  |
| Sputum |  |  | 0.174 | 0.676 |
| Yes | 46(54.1) | 30(58.8) |  |  |
| No | 34(40) | 19(37.3) |  |  |
| Shortness of breath |  |  | 0.006 | 0.936 |
| Yes | 68(80) | 41(80.4) |  |  |
| No | 16(18.8) | 10(19.6) |  |  |
| Complication |  |  | 10.444 | 0.005 |
| No | 25(29.4) | 25(49) |  |  |
| One | 30(35.3) | 6(11.8) |  |  |
| Two or Two plus | 18(21.2) | 14(27.5) |  |  |
| Stabilization therapy |  |  | 4.254 | 0.119 |
| Inhalation of ICS-containing drugs | 44(51.8) | 32(62.7) |  |  |
| Inhalation of ICS-free drugs | 23(27.1) | 15(29.4) |  |  |
| Non-inhalation of drugs | 18(21.2) | 4(7.8) |  |  |
| Treatment |  |  | 9.102 | 0.296 |
| ICS | 2(2.4) |  |  |  |
| ICS+LABA | 19(22.4) | 8(15.7) |  |  |
| ICS+LAMA |  | 1(2) |  |  |
| LABA+LAMA | 15(17.6) | 8(15.7) |  |  |
| ICS+LABA+LAM | 20(23.5) | 20(39.2) |  |  |
| Inhalation + other oral medications | 2(2.4) | 2(3.9) |  |  |
| LAMA | 5(5.9) | 3(5.9) |  |  |
| LABA | 3(3.5) | 4(7.8) |  |  |
| No inhaled medication | 7(8.2) | 1(2) |  |  |
| Number of acute exacerbations 1 year prior to enrollment |  |  | 2.934 | 0.406 |
| Never | 62(72.9) | 37(72.5) |  |  |
| Once | 3(3.5) | 5(9.8) |  |  |
| Twice | 3(3.5) | 3(5.9) |  |  |
| Three times and more | 1(1.2) |  |  |  |
| mMRC |  |  | 12.13 | 0.011 |
| 0 | 20(23.5) | 4(7.8) |  |  |
| 1 | 48(56.5) | 24(47.1) |  |  |
| 2 | 12(14.1) | 14(27.5) |  |  |
| 3 | 4(4.7) | 5(9.8) |  |  |
| 4 |  | 2(3.9) |  |  |
| SGRQ（in the end） | 26.2±17.37 | 36.5±15.78 | -2.456 | 0.014 |
| FEV1/FVC | 65.9±13.01 | 48.9±10.26 | -6.77 | ＜0.001 |
| FEV1%pre | 71.3±10.94 | 36.3±9.99 | -9.743 | ＜0.001 |

**Supplementary Table 4** Correlation Analysis Between Pulmonary Function and Emphysema in COPD Patients and Healthy Individuals

|  | | | Pulmonary Emphysema | | |
| --- | --- | --- | --- | --- | --- |
|  |  |  | Bothlungs | Rightlung | Leftlung |
| FEV1/FVC | COPD | Correlation coefficient r | -0.502 | -0.517 | -0.458 |
|  |  | p value | 0.000 | 0.000 | 0.000 |
|  | Healthy people | Correlation coefficient r | -0.057 | -0.064 | -0.049 |
|  |  | p value | 0.591 | 0.545 | 0.642 |
| FEV1pred | COPD | Correlation coefficient r | -0.370 | -0.377 | -0.345 |
|  |  | p value | 0.000 | 0.000 | 0.000 |
|  | Healthy people | Correlation coefficient r | 0.071 | 0.047 | 0.087 |
|  |  | p value | 0.503 | 0.656 | 0.410 |

Supplementary Discussion：Extended interpretation and cross-disease model relevance
